# MCM10: An effective treatment target and a prognostic biomarker in patients with uterine corpus endometrial carcinoma

**DOI:** 10.1111/jcmm.17772

**Published:** 2023-05-29

**Authors:** Junyu Chen, Shan Wu, Junwei Wang, Chunying Han, Lijing Zhao, Kang He, Yan Jia, Manhua Cui

**Affiliations:** ^1^ Department of Gynecology and Obstetrics The Second Hospital of Jilin University Changchun China; ^2^ Department of Reproductive Endocrinology, Key Laboratory of Reproductive Genetics (Ministry of Education) Women's Hospital, Zhejiang University, School of Medicine Hangzhou China; ^3^ Department of Obstetrics and Gynecology The First Hospital of Jilin University Changchun China; ^4^ Third Department of Gynecological Oncology Jilin Cancer Hospital Changchun China; ^5^ Department of Rehabilitation, School of Nursing Jilin University Changchun China

**Keywords:** MCM10, OS prediction model, TCGA, uterine corpus endometrial carcinoma, variation

## Abstract

Molecular profiling has been applied for uterine corpus endometrial carcinoma (UCEC) management for many years. The aim of this study was to explore the role of MCM10 in UCEC and construct its overall survival (OS) prediction models. Data from TCGA, GEO, cbioPotal and COSMIC databases and the methods, such as GO, KEGG, GSEA, ssGSEA and PPI, were employed to bioinformatically detect the effects of MCM10 on UCEC. RT‐PCR, Western blot and immunohistochemistry were used to validate the effects of MCM10 on UCEC. Based on Cox regression analysis using the data from TCGA and our clinical data, two OS prediction models for UCEC were established. Finally, the effects of MCM10 on UCEC were detected in vitro. Our study revealed that MCM10 was variated and overexpressed in UCEC tissue and involved in DNA replication, cell cycle, DNA repair and immune microenvironment in UCEC. Moreover, silencing MCM10 significantly inhibited the proliferation of UCEC cells in vitro. Importantly, based on MCM10 expression and clinical features, the OS prediction models were constructed with good accuracy. MCM10 could be an effective treatment target and a prognostic biomarker for UCEC patients. The OS prediction models might help establish the strategies of follow‐up and treatment for UCEC patients.

## 1 INTRODUCTION

Uterine corpus endometrial carcinoma (UCEC) is the sixth most common malignancy in women, causing 417,000 new cases diagnosed worldwide in 2020.^1^ The incidence of UCEC is rising in developed countries due to the increased prevalence of obesity and metabolic syndrome, while the mortality rates of patients from underdeveloped countries are higher than those from developed countries because of poor medical care.^2^ This phenomenon poses a serious threat to women's health globally. Although most of the patients with early phase UCEC could be cured by hysterectomy and adjuvant radiotherapy, women with advanced UCEC still have a poor prognosis, with five‐year survival rates of approximating 48% in stage III and 15% in stage IV.^3^ The combination of carboplatin and paclitaxel, the first line of advanced UCEC, only results in 13 months of progress‐free survival.^4^ Unfortunately, within the last three decades, there were only a few drugs approved for use in advanced UCEC by the US Food Drug Administration (FDA). Chemotherapy remains a standard management for patients with advanced or recurrent UCEC.^4^, ^5^

Molecular profiling has been used to manage these advanced and recurrent UCEC patients for many years. For example, progesterone receptor and oestrogen receptor status have been used to evaluate whether patients can be treated with hormone therapy. However, even in some patients with positive progesterone receptor and oestrogen receptor, single‐agent aromatase inhibitors only achieved 10% response rate.^2^ The use of mTOR inhibitor yielded a response rate of merely 10%.^6^ Hence, it is still urgent to find new targets and further understand its molecular biology in UCEC, and these efforts will contribute to effective targeted chemotherapeutic strategies in the future.

Minichromosome maintenance 10 replication initiation factor (MCM10) gene is a protein‐coding gene located on chromosome region 10p13. MCM10 was first found to involve in the initiation process of DNA replication in yeast.^7^ It has been revealed that MCM10 is highly expressed in bone marrow, lymph node and appendix, but relatively low in endometrium tissue.^8^ In recent years, MCM10 has been proved to promote the DNA elongation process through inhibiting the activity of Cdc45/MCM2‐7/GINS complex and ensuring the stability of replication fork.^9^, ^10^ These properties allow MCM10 to play an important role in cell proliferation, and even in cellular immortalization. Overexpression of MCM10 has been found in various cancers, including ovarian cancer,^11^ breast cancer^12^ and prostate cancer^13^ and is associated with poor prognosis. A pan‐cancer analysis based on GEPIA database showed that MCM10 mRNA expression was overexpressed in UCEC as compared with normal endometrium.^11^ Considering the close relationship between MCM10 and various malignant tumours, we believe that MCM10 may also be associated with the development of UCEC and can be an effective prognosis biomarker.

In this study, data from breast cancer, cervical cancer, uterine sarcoma and UCEC in The Cancer Genome Atlas (TCGA) database were used to screen novel biomarkers, and MCM10 was identified to be an effective indicator to predict UCEC prognosis. Subsequently, based on the bioinformatic analysis, we explored the potential function of MCM10 in the development of UCEC and established an overall survival (OS) prediction model for UCEC. Furthermore, in order to validate the effects of MCM10 in UCEC and the reliability of this model, clinical data and pathological sections stained by immunohistochemical staining were employed to construct our clinical data‐based OS prediction model for UCEC. Finally, the potential function of MCM10 in the development of UCEC was verified in vitro.

## 2 MATERIALS AND METHODS

### 2.1 The screening of biomarkers predicting the prognosis of UCEC

The data sets of breast cancer (BRCA), cervical squamous cell carcinoma and endocervical adenocarcinoma (CESC), uterine carcinosarcoma (UCS) and UCEC from TCGA database were employed to screen the biomarkers predicting the prognosis of UCEC. The R package ‘DESeq2’ was used to obtain four differentially expressed genes (DEGs) between tumour and normal tissue (tumour vs. normal) based on the data sets of BRCA, CESC, UCS and UCEC, respectively. The Log_2_ Fold Change (LogFC) > 3 and adjusted *p*‐value <0.05 were considered as the cut‐off of DEGs. After taking the intersection of the four DEGs, the top 20% overexpression genes in UCEC were selected to be further screened by Kaplan–Meier analysis in UCEC. Finally, the gene, MCM10, has not been reported to be associated with UCEC were selected in this study.

The differential expression data of MCM10 in unpaired and paired samples are RNA‐seq data from TCGA UCEC project and GTEx database. The data were uniformed by the Toil process.^14^ All final analyses based on TCGA were performed using data in TPM format. The differential analysis data for MCM10 in UCEC based on the data set GSE17025 was employed to further validate that MCM10 mRNA was overexpressed in UCEC tissues.

### 2.2 Single‐gene differential analysis and correlation analysis of MCM10

Single‐gene differential analysis and single‐gene correlation analysis of MCM10 in UCEC project based on TCGA database were performed using the DESeq2 package [version 1.26.0] and STAT package [version 3.6.3], respectively.^15^ The UCEC samples were divided into high‐expression and low‐expression groups based on MCM10 median expression level. Volcano plots were drawn based on the results of single‐gene differential analysis using the ggplot2 package [version 3.3.3]. The ∣LogFC∣ > 1 and adjusted *p*‐value <0.05 were considered as the cut‐off of the DEGs. STRING database was used to visualize the analysis of DEGs,^16^ and protein–protein interaction (PPI) of DEGs was performed using Cytoscape software network analysis, and then the HUB genes were identified using the MCODE plugin. Finally, the genes were sorted by ‘Pearson value’ in descending order based on single‐gene correlation analysis, and the genes whose correlations were in the top 50 were extracted. The single‐gene co‐expression heat map of MCM10 was drawn by ggplot2 [version 3.3.3] package using the top 50 genes, MCM gene family members (MCMs), including MCM2, MCM3, MCM4, MCM5, MCM6, MCM7, MCM8, and MCM9, and MKI67 gene.

### 2.3 Variation analysis of MCM10 in UCEC

The variation types and frequency of MCMs and MCM10 in UCEC were obtained from the cBioPortal database (http://www.cbioportal.org/). ‘Uterus’ and ‘Endometrial Carcinoma’ were chosen as the object. A total of 509 endometrial cancer cases from TCGA database was chosen as the sample. The mutation types and frequency of MCM10 in UCEC were obtained from the Catalogue of Somatic Mutations in Cancer (COSMIC) (https://cancer.sanger.ac.uk). Endometrial in ‘tissue distribution’ and ‘mutation distribution’ were chosen.

### 2.4 Functional enrichment analysis

Gene set enrichment analysis (GSEA) was used to explore potential signalling pathways based on the differential expression analysis (MCM10 high‐expression vs. MCM10 low‐expression samples) in TCGA UCEC project. And c2.cp.v7.2. symbols. gmt was used as the reference gene set. Adjusted *p*‐value <0.05 was considered as significantly enriched. After screening the DEGs based on the cut‐off (∣LogFC > 1∣ and adjusted *p*‐value <0.05), Gene Ontology (GO), Kyoto Encyclopedia of Genes and Genomes (KEGG) analysis were performed to further enrich the pathways related to MCM10 in UCEC with the R package ‘clusterProfiler’ and ‘org.Hs.eg.db’. Adjusted *p*‐value <0.05 was considered as significantly enriched.

### 2.5 Detection of immune cell subtypes in MCM10 high‐expression and MCM10 low‐expression UCEC samples

The analysis of immune cell subtypes in TCGA UCEC project was analysed using the GSVA package [version 1.34.0] and single‐sample gene set enrichment analysis (ssGSEA) algorithm.^17^ The biomarkers of 24 immune cells were obtained from a previous study.^18^ GSVA package [version 1.34.0] was used to analysis and visualize the relative proportion of immune cell subtypes between the MCM10 high‐expression and MCM10 low‐expression groups.

### 2.6 Analysis of the correlation between MCM10 expression and clinical features of patients with UCEC

The clinical features of patients with UCEC were obtained from TCGA UCEC project and the Second Hospital of Jilin University, which was used to analyse their correlation with MCM10 expression in UCEC samples. Then, ggplot2 [version 3.3.3] was employed to visualize the results. A receiver operating characteristic (ROC) curve that could demonstrate the ability of MCM10 protein to distinguish endometrial cancer from normal endometrium was drawn to further verify the correlation between MCM10 and UCEC using the pROC package [version 1.17.0.1]. Furthermore, the degree of correlation between the clinical characteristics of UCEC and MCM10 was evaluated by logistics regression, which was visualized by forest plots.

### 2.7 Analysis of the correlation between MCM10 expression and prognosis of patients with UCEC

The survival data of UCEC patients from TCGA database were used to analyse the correlation between the mRNA expression of various genes (including HOXB13, PITX1, MYBL2, IGF2BP1, CDC20, NMU, CA9, UBE2C, PKMYT1, BIRC5, MCM10 and MMP1) and the prognosis of patients with UCEC using survival package [version 3.2–10] based on Kaplan–Meier method, and the survminer package [version 0.4.9] was used to plot Overall Survival (OS) curves.

Furthermore, univariate Cox regression was used to screen out the risk factors affecting OS from the clinical characteristics and MCM10 expression levels of UCEC patients. After being screened by univariate Cox regression with a cut‐off of *p*‐value <0.1, the significant factors were then included in a multivariate Cox regression model to find the independent risk factors for the OS of UCEC patients. The analysis was performed using the survival package [version 3.2–10], which was visualized by forest plots.

Based on the results of multivariate Cox regression, the rms package [version 6.2–0] and survival package [version 3.2–10] were employed to construct the prognostic prediction model predicting 4‐, 6‐ and 8‐year OS, which was visualized by nomograms. Moreover, the calibration plots were drawn using the rms package [version 6.2–0] and survival package [version 3.2–10] to check the accuracy of the prediction models.

### 2.8 Specimens

The present study was approved by the Ethics Committee of the School of Nursing, Jilin University (Changchun, China). The patients were informed and agreed to participate in this study. Paraffin‐embedded specimens were collected at the Second Hospital of Jilin University from 119 patients with UCEC and 15 normal controls who were diagnosed between October 2013 and December 2019. The inclusion criteria were as follows: (i) Initially diagnosed with UCEC and treated with standard surgery and/or radiotherapy and/or chemotherapy according to the International Federation of Gynaecology and Obstetrics (FIGO) stage and pathological type of individual patients; (ii) the diagnosis was determined as UCEC by an experienced pathologist; (iii) complete follow‐up data were available. The exclusion criteria were as follows: (i) Individual history of other malignant tumours; (ii) the patients had undergone preoperative radiotherapy, chemotherapy or hormonotherapy; and (iii) secondary uterine tumour. The available clinical/pathological data are shown in Table 1. The median follow‐up for patients with UCEC was 43 months, ranging between 7 and 107 months.

**TABLE 1:** Associations between MCM10 protein expression and clinical features of patients with UCEC.

Clinical features	Number of cases	Low expression of MCM10, number (%)	High expression of MCM10, number (%)	*p*
Age, *n* (%)^a^				
<55	53	27 (22.7%)	26 (21.8%)	0.79
> = 55	66	32 (26.9%)	34 (28.6%)	
Menopause status, *n* (%)^a^				
No	39	15 (12.6%)	24 (20.2%)	0.09
Yes	80	44 (37.0%)	36 (30.3%)	
Diabetes, *n* (%)^a^				
No	99	50 (42.0%)	49 (41.2%)	0.653
Yes	20	9 (7.6%)	11 (9.2%)	
Histological type, *n* (%)^b^				
Endometrioid	102	51 (42.9%)	51 (42.9%)	0.710
Serous	4	1 (0.8%)	3 (2.5%)	
Mix	13	7 (5.9%)	6 (5.0%)	
Histologic grade, *n* (%)^a^				
G1	44	32 (27.6%)	12 (10.3%)	< 0.001^c^
G2	60	18 (15.5%)	42 (36.2%)	
G3	12	6 (5.2%)	6 (5.2%)	
Tumour invasion (%), *n* (%)^a^				
<50	80	45 (37.8%)	35 (29.4%)	0.037*
> = 50	39	14 (11.8%)	25 (21.0%)	
Clinical stage, *n* (%)^b^				
Stage I	81	51 (42.9%)	30 (25.2%)	< 0.001^c^
Stage II	13	2 (1.7%)	11 (9.2%)	
Stage III	20	4 (3.4%)	16 (13.4%)	
Stage IV	5	2 (1.7%)	3 (2.5%)	
Lymphatic metastasis, *n* (%)^b^				
No	78	38 (40.0%)	40 (42.1%)	0.066
Yes	17	4 (4.2%)	13 (13.7)	
Primary therapy outcome, *n* (%)^b^				
PR	8	1 (0.8%)	7 (6.0%)	0.061
CR	111	58 (48.7%)	53 (44.5%)	

^a^ The results were analysed by Chi‐squared test.

^b^ The results were analysed by Fisher's test.

^c^ *p* < 0.01.

### 2.9 Cell culture and stably transfected cell line development

The human UCEC cell lines Ishikawa and HEC‐1‐A (iCell Bioscience Inc.) were cultured at 37 °C in a humidified atmosphere with 5% CO_2_. The interference sequences were as follows: shMCM10, 5'‐CGGCGACGGTGAATCTTAT‐3' and shScramble, 5'‐GTATAAGTCAACTGTTGAC‐3'. The lentiviruses used in this study were packaged using the three plasmids packaging system. The lentiviral plasmids (pLKO.1‐shMCM10 or pLKO.1‐Scramble) with two packaging plasmids, including PMD2.G (Fenghui) and psPAX2 (Fenghui) and Lipofectamine™ 3000 transfection reagent (Thermo) were added into HEK‐293 T cells (iCell Bioscience Inc.). After 48 and 72 h, the medium was collected and filtered using 0.22 𝜇m filter. To generate stably transfected cell lines, Ishikawa and HEC‐1‐A cells were seeded into six‐well plates (300,000 cells/well). 1 mL medium containing the above lentiviral was added into each well. The cells were selected in medium containing puromycin (2 μg/mL) and maintained in medium containing puromycin (1 μg/mL). Puromycin selection was continued for 1 week prior to harvesting cells for downstream analysis.

### 2.10 Immunohistochemistry

Immunohistochemical staining was performed as our previous study.^19^ The sections were stained with rabbit anti‐MCM10 antibody (1:100; Affinity) and anti‐MKI67 antibody (1:400; Servicebio). Goat anti‐rabbit IgG conjugated with horseradish peroxidase (1:200; Servicebio) was used as the secondary antibody. Histological images were captured under a light microscope (Motic) and assessed using Image‐Pro Plus 6.0 software (Media Cybernetics, Inc.). The results are presented as the mean optical density (MOD) values.

### 2.11 RT‐PCR

Real‐time PCR was used to detect the transcription levels of MCM10 using the SYBR Green qPCR kit (TransGen) according to the manufacturer's instructions, and 𝛽‐actin was used as an internal control gene. The following primers were used: MCM10 gene forward, 5'‐GGAGAGAACAACTTGCCTATCTG‐3' and reverse, 5'‐AGTAGCGCTCCTGCATCTCA‐3′; 𝛽‐actin gene forward, 5'‐GTGGCCGAGGACTTTGATTG‐3′ and reverse, 5'‐CCTGTAACAACGCATCTCATATT‐3′. The mRNA expression levels were quantified using 2−ΔΔCq method.^19^

### 2.12 Western blot

A total of 15 μg soluble proteins were subjected to each lane of a 4%–20% Precast BIS‐Tris Gel (Absin). Separated proteins were electrophoretically transferred onto polyvinylidene difluoride membranes (Thermo). The membranes were incubated with primary antibodies, including anti‐MCM10 (1:1000; Affinity) and anti‐GAPDH (1:20,000; Bioworld) and secondary‐HRP antibodies (1:20,000; Bioworld). The protein levels were evaluated under an imaging densitometer (Clinx). The assay was repeated three times independently.

### 2.13 Cell Counting Kit‐8 (CCK‐8) assay

Ishikawa, HEC‐1‐A, Ishikawa‐shScramble, HEC‐1‐A‐shScramble, shikawa‐shMCM10 and HEC‐1‐A‐shMCM10 cells were seeded into 96‐well plates (3000 cells/well). Subsequently, 24, 48 and 72 h later, CCK‐8 reagent (10 μL/well; Bioss) was added into each well. The absorbance of each well was measured at 450 nm using a microplate reader (E0226; Detie).^20^

### 2.14 Colony formation assay

The indicated cells were seeded onto 6‐well plates at a density of 100 cells/well. 10 days later, the formation of typical clone of the cells was observed. The cells were fixed with methanol and stained with 10% Giemsa (Biotopped). The number of visible colonies was counted to evaluate the colony formation ability of cells.^21^

### 2.15 Immunofluorescence

The indicated cells were seeded on six‐well plates at 2 × 10^6^ cells/well. Standard immunofluorescence procedures were carried out as a previous study.^22^ Rabbit anti‐MKI67 (Servicebio) antibody was used as the primary antibody. Goat anti‐Rabbit IgG (H + L) conjugated with Cy5 (Servicebio) was used as the secondary antibody. The cover slips were observed using a fluorescence microscope (Olympus). Blue represented the nucleus stained with DAPI, and red represented MKI67 protein.

### 2.16 Statistical analysis

ssGSEA was used for the algorithm of immune infiltration. Statistical analyses were performed with SPSS 23.0 or R version 3.6.3, the differences among the groups were compared using one‐factor analysis of variance (anova) followed by Dunnett's post hoc test, Kruskal–Wallis test or student's *t* test. Crosstab data were compared by Chi‐squared or Fisher's test. Differences in *p* < 0.05 were considered statistically significant. All results were repeated three times independently.

## 3 RESULTS

### 3.1 MCM10 might be related to the development and prognosis of UCEC patients

**FIGURE 1:**
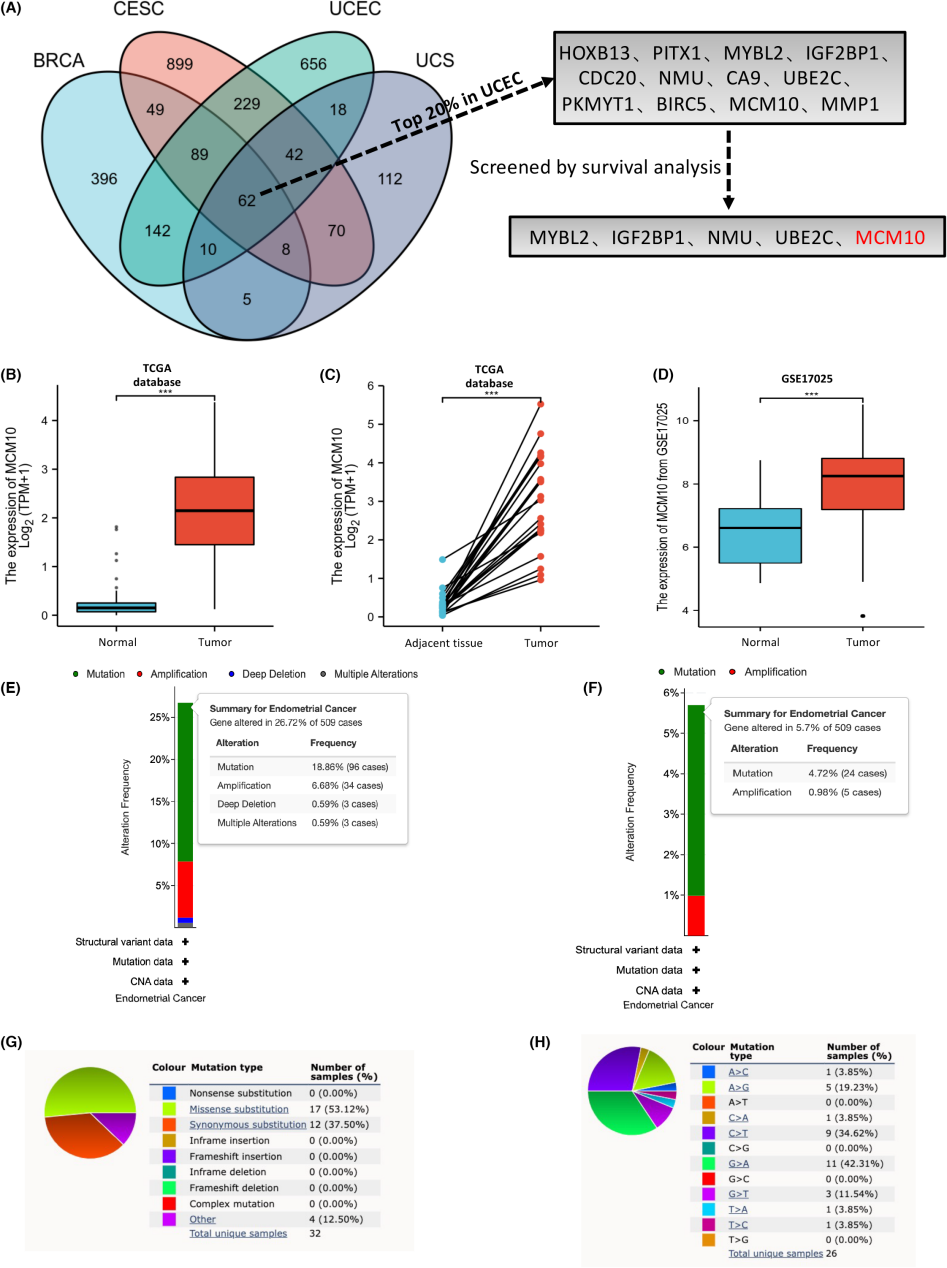
Expression and variation of MCM10 in UCEC tissue. (A) The process of screening MCM10 is showed by a Venn diagram; (B, C) the expression of MCM10 mRNA in UCEC tissue, adjacent tissue of UCEC and normal endometrium tissue based on TCGA database; (D) the expression of MCM10 mRNA in UCEC and normal endometrium tissue based on GEO database; (E) variation types and frequency of MCMs in UCEC in cBioPortal database; (F) variation types and frequency of MCM10 in UCEC in cBioPortal database; (G, H) the mutation types of MCM10 in UCEC in COSMIC database. ***p < 0.001.

As shown in Figure 1A and Table S1, 62 upregulated genes (LogFC > 3) were identified through the comparison of the DEGs (cancer tissue vs normal tissue) of BRCA (Table S2), CESC (Table S3), UCEC (Table S4) and UCS (Table S5) based on TCGA database. Among them, the top 20% of genes with high expression in UCEC (Table S6) were as follows: HOXB13, PITX1, MYBL2, IGF2BP1, CDC20, NMU, CA9, UBE2C, PKMYT1, BIRC5, MCM10 and MMP1 (Figure 1A). These genes were then selected for survival analysis in UCEC. Finally, MYBL2, IGF2BP1, NMU, UBE2C and MCM10 were found to be associated with the OS of UCEC patients (*p* < 0.05) (Figure 1A and Figure S1). To the best of our knowledge, only MCM10 has not been reported to be associated with the development and prognosis of UCEC. Thus, we selected MCM10 as a new target to explore its relationship with UCEC. Based on TCGA database, we found that the expression of MCM10 mRNA was upregulated in UCEC tissues, both compared with normal tissues (Figure 1B) and adjacent tissues (Figure 1C). Moreover, the result from GSE17025 database also testified that MCM10 mRNA was overexpressed in UCEC tissues as compared to that in normal control tissues (Figure 1D).

### 3.2 Variation of MCM10 in UCEC

The results from the cBioPortal database showed that 26.72% (136/509) of UCEC patients had variation of MCMs (Figure 1E), of which MCM10 accounted for 5.7% (29/509), and mutation was the most frequent variation (Figure 1F). Therefore, COSMIC database was used to further explore the types of mutation. The results showed that missense substitution occurred in 53.12% of the UCEC samples and synonymous substitution occurred in 37.50% of the UCEC samples (Figure 1G). Furthermore, the base substitutions were mainly G > A (42.31%), C > T (34.62%) and A > G (19.23%) (Figure 1H).

### 3.3 Enrichment analysis of the MCM10 expression phenotype

**FIGURE 2:**
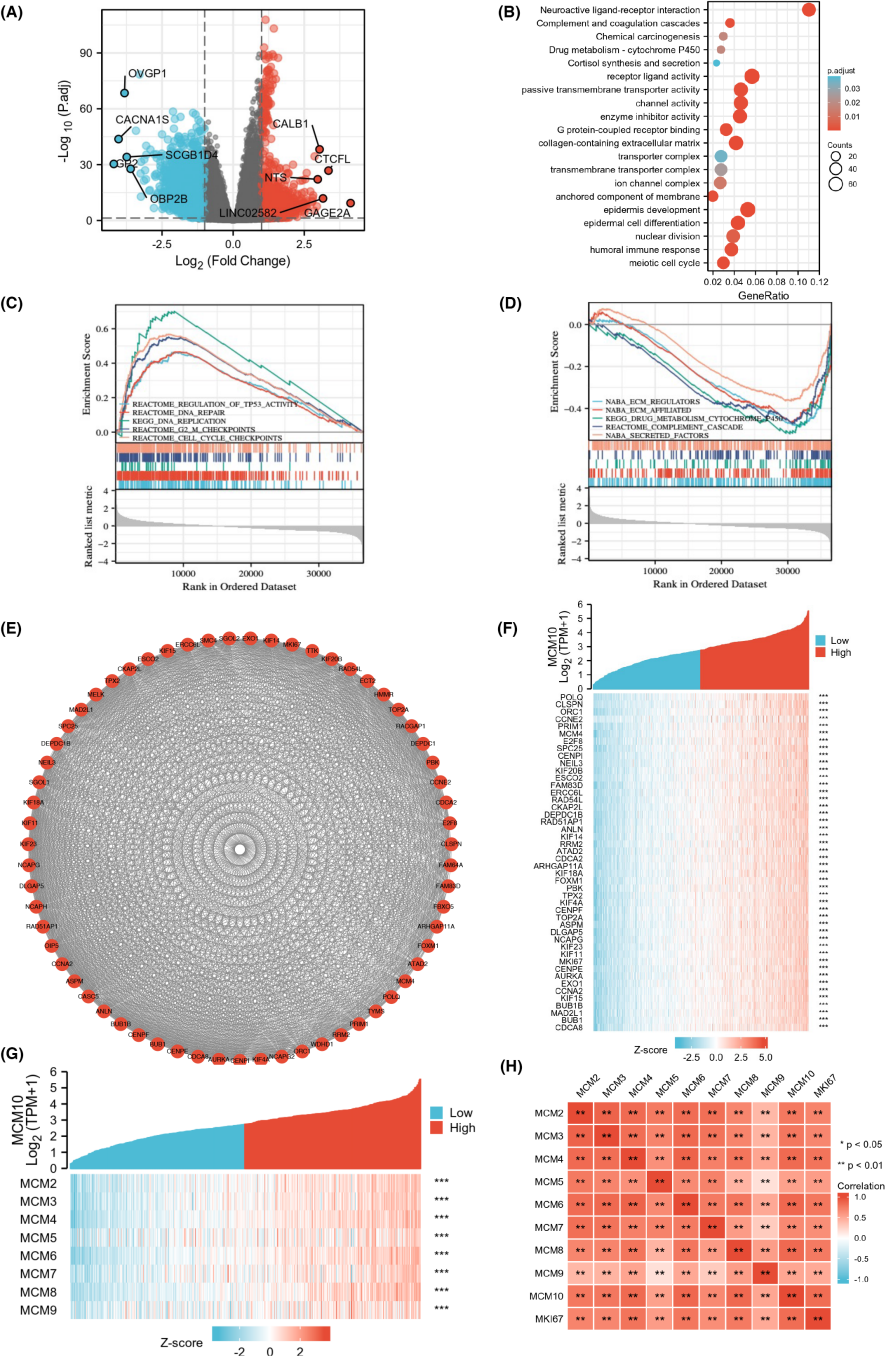
Gene function analysis of MCM10 based on TCGA database. (A) Volcano plot of the DEGs analysis, and the top ten genes with the greatest differences were marked; (B) KEGG and GO enrichment analysis of DEGs (∣LogFC∣ > 1, adjusted *p*‐value <0.05); (C, D) GSEA enrichment analysis of DEGs; (E) PPI analysis of DEGs; (F) The top 50 genes correlated with MCM10 was showed in the heat map; (G) the correlation between MCMs and MCM10 was showed in the heat map; (H) the correlation between MCMs and MKI67 was shown in the heat map. **p < 0.01, ***p < 0.001.

Based on TCGA database, a total of 3966 DEGs (|LogFC| > 1, adjusted *p*‐value <0.05) were identified between UCEC samples with high expression and low expression of MCM10 (Table S7). In this regard, the samples with low MCM10 expression were considered as controls. As shown in the volcano plot (Figure 2A), 1616 genes (LogFC > 1, adjusted *p*‐value <0.05) were considered as high‐expression genes, and 2350 genes (LogFC < −1, adjusted *p*‐value <0.05) were considered as low‐expression genes in the UCEC samples with high MCM10 expression. Twenty related signalling pathways for the 3966 DEGs were enriched and showed by GO and KEGG analysis. Remarkably, we found that the enrichment pathways were mainly associated with humoral immune response, nuclear division, meiotic cell cycle, collagen‐containing extracellular matrix, G protein‐coupled receptor binding, channel activity, enzyme inhibitor activity, passive transmembrane transporter activity and neuroactive ligand–receptor interaction (Figure 2B and Table S8).

Moreover, in order to explore the relevant pathways of MCM10 in UCEC more comprehensively, the data of 3966 DEGs were further used to perform GSEA. The GSEA results indicated that the regulation of TP53 activity, DNA repair, DNA replication, G2/M checkpoints and cell cycle checkpoints were enhanced in the samples with high MCM10 mRNA expression (Figure 2C), whereas some pathways were inhibited, including ECM regulators, ECM affiliated, drug metabolism cytochrome, complement cascade and secreted factors (Figure 2D). More GSEA results were shown in Table S9.

### 3.4 Analysis of genes associated with MCM10 in UCEC

As shown in Figure 2E, 63 HUB genes related to MCM10 in UCEC were identified in the PPI network using MCODE plugin. The correlation between MCM10 mRNA expression and the top 50 of these HUB genes was presented in Figure 2F. Moreover, the correlation between MCM10 mRNA expression and MCM family genes, including MCM2, MCM3, MCM4, MCM5, MCM6, MCM7, MCM8 and MCM9, was shown in Figure 2G. Finally, we found that most of MCMs mRNA expression was positively correlated with MKI67 mRNA expression (*r* > 0.7, *p* < 0.01) (Figure 2H).

### 3.5 The effects of MCM10 changes on immune cell infiltration in UCEC

To detect the effects of MCM10 changes on the tumour microenvironment, immune infiltration analysis was performed using the ssGSEA method. As shown in Figure S2A and Table S10, the proportion of T help2 (Th2) cells was significantly higher in MCM10 high‐expression group than that in the low‐expression group. The proportions of natural killer (NK) CD56 bright cells, plasmacytoid dendritic cell (pDC) cells, immature dendritic cell (iDC cells), NK cells and eosinophils were significantly lower in MCM10 high‐expression group than that in the low‐expression group. Furthermore, we found that MCM10 was positively correlated with Th2 cells (*r* = 0.592, *p* < 0.001), while negatively correlated with NK CD56bright cells (*r* = − 0.573, *p* < 0.001), pDC cells (*r* = −0.478, *p* < 0.001), iDC cells (*r* = −0.357, *p* < 0.001), NK cells (*r* = −0.327, *p* < 0.001) and eosinophils (*r* = −0.305, *p* < 0.001) (Figure S2B,C).

### 3.6 Analysis of the association between MCM10 expression and the clinical features of patients with UCEC

**FIGURE 3:**
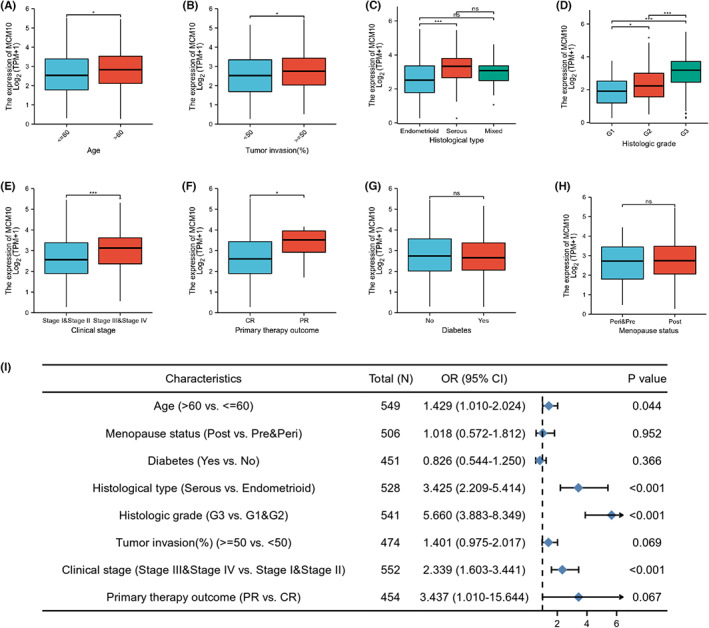
Correlation analysis between MCM10 and clinical features based on TCGA database. The correlation between MCM10 mRNA expression and clinical features, including (A) age, (B) tumour invasion, (C) histological type, (D) histologic grade, (E) clinical stage, (F) primary therapy outcome, (G) diabetes and (H) menopause status; (I) the risk factors for MCM10 mRNA high expression were shown in the forest plot based on logistic regression analysis. *p < 0.05 and ***p < 0.001.

To further demonstrate the effect of MCM10 expression on UCEC development in patients, the association between MCM10 expression and clinical features of patients with UCEC was evaluated using TCGA database and the clinical data from the Second Hospital of Jilin University. The clinical baseline information tables of TCGA database (Table S11) and our clinical data (Table 1) were constructed based on the expression level of MCM10. Through bioinformatic analysis based on TCGA database, we found that age (Figure 3A), tumour invasion (Figure 3B), histological type (Figure 3C), histologic grade (Figure 3D), clinical stage (Figure 3E) and primary therapy outcome (Figure 3F) were identified to be associated with the expression of MCM10 mRNA. No significant association was observed between MCM10 mRNA expression and diabetes (Figure 3G) or menopause status of the patients (Figure 3H). More specifically, the mRNA expressions of MCM10 were higher in the groups of age > 60, tumour invasion > = 50%, serous type, grade 2 (G2), grade 3 (G3), stage III and IV and part response (PR) compared with that in the control groups (*p* < 0.05). Furthermore, the logistics regression analysis showed that age > 60, serous type, G3 and stage III and IV were risk factors of MCM10 mRNA high expression in UCEC (Figure 3I).

**FIGURE 4:**
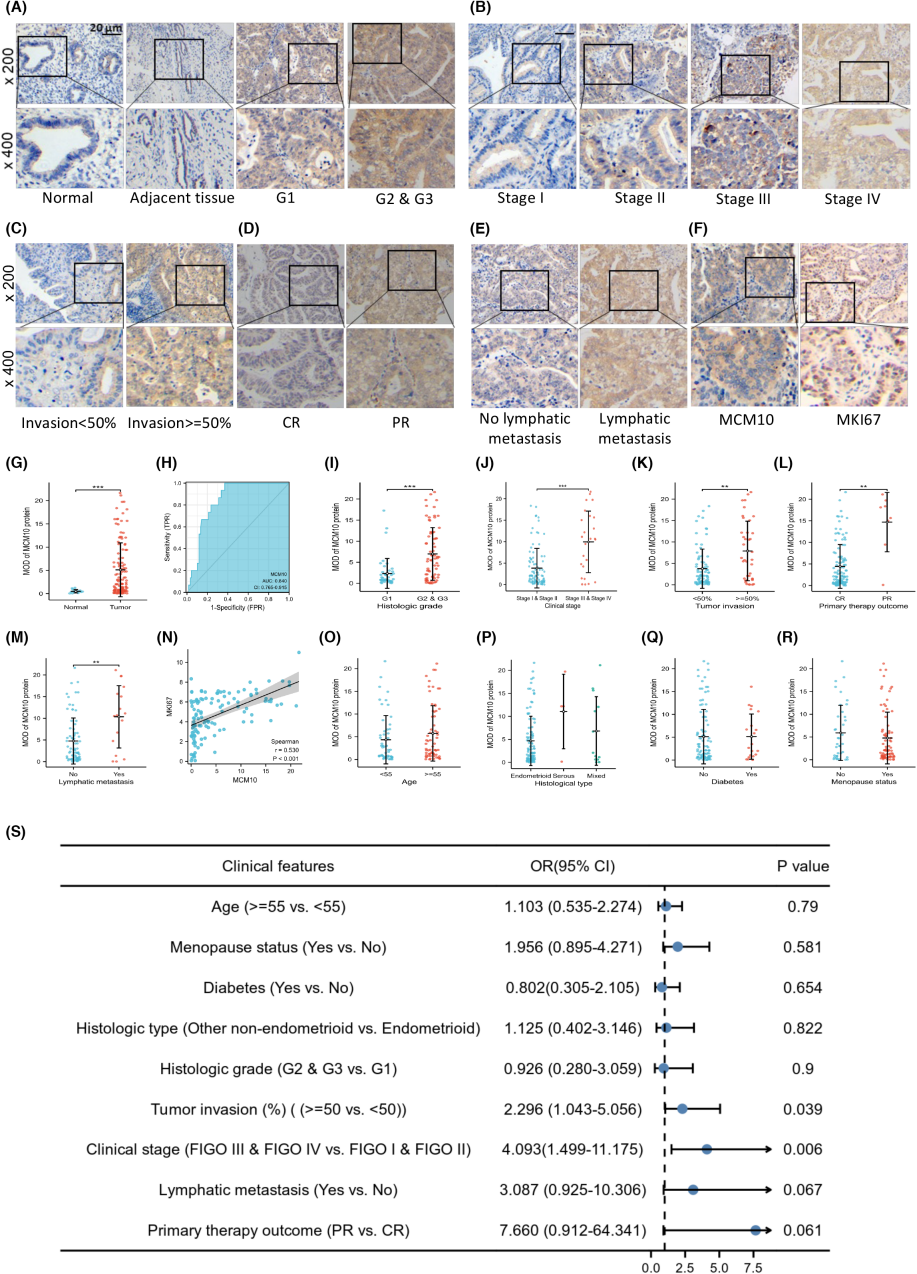
Correlations between MCM10 protein expression and clinical features were validated by clinical samples. The significant changes of MCM10 protein expression in (A, G, H, I) Normal tissues, adjacent tissues of UCEC, G1 UCEC tissues and G2 and G3 UCEC tissues, (B, J) UCEC tissues of patients with FIGO I, FIGO II, FIGO III and FIGO IV, (C, K) UCEC tissues of patients with invasion <50% and invasion > = 50%, (D, L) UCEC tissue of patients with complete response (CR) and partial response (PR), and (E, M) UCEC tissues of patients with lymphatic metastasis and no lymphatic metastasis; (F, N) MCM10 expression was positively correlated with MKI67; the expression of MCM10 protein in (P) UCEC tissues of Endometrioid, Serous, and Mixed histological types, (Q) UCEC tissues of patients with diabetes and no diabetes and (R) UCEC tissues of patients with menopause status and pre‐menopausal status; (S) the risk factors for MCM10 protein high expression were shown in the forest plot based on logistics regression analysis; *p < 0.05, **p < 0.01, and ***p < 0.001.

The analysis based on our clinical data showed that MCM10 protein expression significantly (*p* < 0.05) increased in UCEC tissues as compared with that in normal or adjacent tissues (Figure 4A,G). The ROC curve showed a well ability of MCM10 protein to distinguish endometrial cancer from normal endometrium (AUC = 0.840, CI: 0.765–0.915) (Figure 4H). Moreover, histologic grade (Figure 4A,I), clinical stage (Figure 4B,J), tumour invasion (Figure 4C,K), primary therapy outcome (Figure 4D,L) and lymphatic metastasis (Figure 4E,M) were identified to be associated with MCM10 protein expression. More specifically, the protein expressions of MCM10 were higher in the groups of G2 and G3, tumour invasion > = 50%, stage III and IV, lymphatic metastasis and PR compared with in the control groups (*p* < 0.05). Interestingly, we found that MCM10 protein expression was positively correlated with MKI67 protein expression (*r* = 0.530, *p* < 0.001) (Figure 4N). No significant association was observed between MCM10 protein expression and age, histological type, diabetes and menopause status of the patients, respectively (Figure 4O–R). Furthermore, the logistics regression analysis showed that tumour invasion > = 50% and stage III and IV were risk factors of MCM10 protein high expression in UCEC (Figure 4S).

### 3.7 Construction, evaluation and visualization of OS prediction models based on the results of multivariate Cox regression in UCEC patients

**FIGURE 5:**
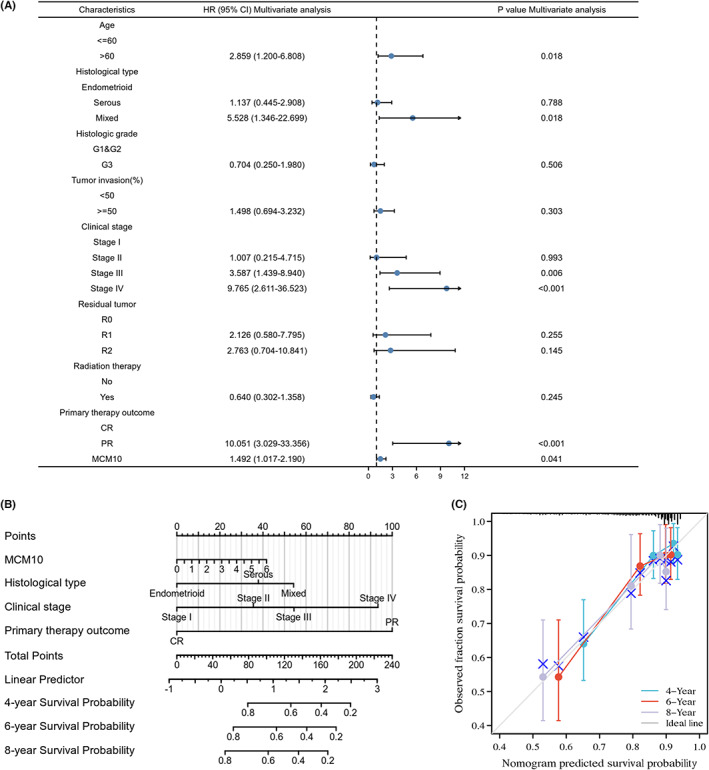
Construction of OS prediction model for UCEC patients based on TCGA database. (A) Results of multivariate Cox regression, which were shown in forest plot; (B) the OS prediction model was visualized by nomogram model; (C) the accuracy of the OS prediction model was evaluated by calibration graph.

As shown in Figure S3, the univariate Cox regression analysis identified that age > 60, serous type, mixed histological type, G3, stage III, stage IV, R2, PR and MCM10 mRNA expression levels were significant risk factors for the OS of patients with UCEC in TCGA database (*p* < 0.05). Radiation therapy was a prevention factor for the OS of UCEC patients (*p* < 0.05). Based on the results of univariate Cox regression analysis, we found that age > 60, mixed histological type, stage III, stage IV, PR and MCM10 mRNA expression levels were independent risk factors for the OS of patients with UCEC in TCGA database (*p* < 0.05) (Figure 5A). As shown in Figure 5B, the OS prediction model included MCM10 mRNA expression level, histological type, clinical stage and primary therapy outcome, which was visualized by the nomogram. The calibration plot of the model showed satisfactory agreement between the prognostic prediction and actual observation (Figure 5C).

**FIGURE 6:**
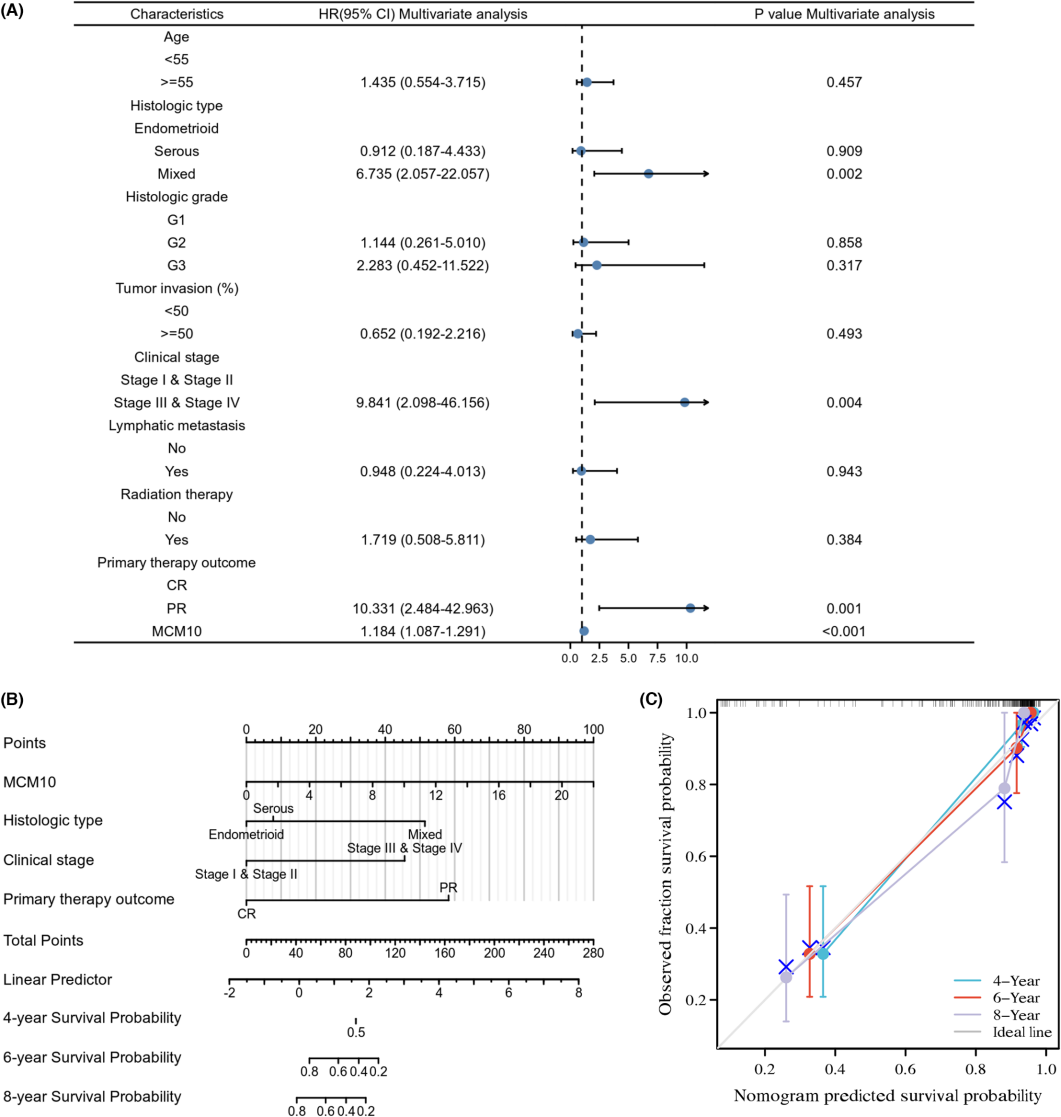
Construction of OS prediction model for UCEC patients based on our clinical data. (A) Results of multivariate Cox regression, which were shown in forest plot; (B) the OS prediction model was visualized by nomogram model; (C) the accuracy of the OS prediction model was evaluated by calibration graph.

As shown in Figure S4, the univariate Cox regression analysis identified that age > =55, serous type, mixed histological type, G2, G3, stage III and stage IV, lymphatic metastasis, PR and MCM10 protein expression level were significant risk factors for the OS of patients with UCEC in our clinical data (*p* < 0.05). Based on the results of multivariate Cox regression analysis, we found that mixed histological type, stage III and stage IV, PR and MCM10 protein expression levels were independent risk factors for the OS of patients with UCEC (*p* < 0.05) (Figure 6A). As shown in Figure 6B, the OS prediction model included MCM10 protein expression level, histological type, clinical stage and primary therapy outcome, which was visualized by the nomogram. The calibration plot of the model showed satisfactory agreement between the prognostic prediction and actual observation (Figure 6C).

### 3.8 Proliferation ability of UCEC cells is inhibited by downregulating MCM10 in vitro

**FIGURE 7:**
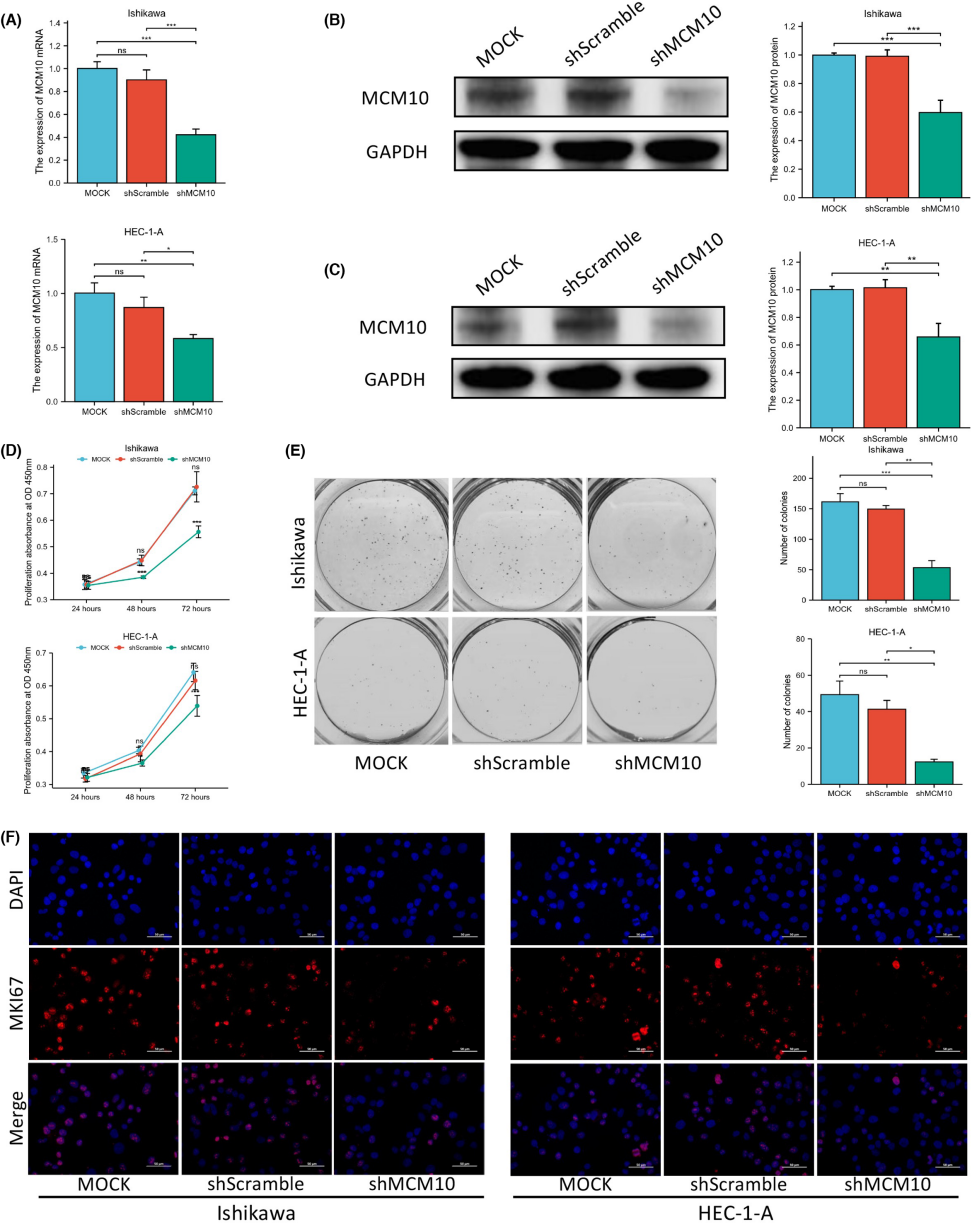
Effects of silencing MCM10 were detected in vitro. (A) RT‐PCR was used to detect the efficiency of MCM10 down‐regulation in Ishikawa and HEC‐1‐A cell lines; Western blot was used to detect the efficiency of MCM10 down‐regulation in (B) Ishikawa and (C) HEC‐1‐A cell lines; (D) CCK‐8 and (E) colony formation assay was used to detect the proliferation ability of Ishikawa and HEC‐1‐A cell lines; (F) immunofluorescence was used to observe the expression of MKI67 protein in Ishikawa and HEC‐1‐A cell lines. Blue represented the nucleus stained with DAPI, and red represented MKI67 protein. *p < 0.05, **p < 0.01, and ***p < 0.001.

The results of RT‐PCR (Figure 7A) and Western blot showed that the expression of MCM10 in Ishikawa (Figure 7B) and HEC‐1‐A (Figure 7C) cells was significantly downregulated after being transfected with shMCM10 (*p* < 0.05). As shown in Figure 7D, from day 2, compared with the Mock and shScramble groups, the cell proliferation of Ishikawa and HEC‐1‐A cells in the shMCM10 groups was remarkably lower (*p* < 0.05). Moreover, the colony ability of Ishikawa and HEC‐1‐A cells in shMCM10 groups was significantly (*p* < 0.05) attenuated compared to the Mock and shScramble groups (Figure 7E). Importantly, the expression of MKI67 significantly decreased in shMCM10 groups as compared with the MOCK and shScramble groups (Figure 7F).

## 4 DISCUSSION

MCMs have been identified to contribute to tumorigenesis and tumour cell proliferation in various cancers.^23^, ^24^ As a member of MCMs, research on MCM10 has been increasing in recent years. Just like other MCMs, the variation of MCM10 gene has been found in many cancers, such as breast cancer, ovarian cancer and gastric cancer.^10^, ^11^, ^25^ Unlike in ovarian cancer where MCM10 gene variation is mainly concentrated on amplification,^11^ the variation of MCM10 is mainly concentrated on mutations in UCEC, which might be a potential mechanism of our finding that both of MCM10 mRNA and protein were overexpressed in UCEC tissue. However, to the best of our knowledge, no studies have explored the role of MCM10 in the development of UCEC. In our study, the expression level of MCM10 was found to be related to multiple clinical features that were associated with the development of UCEC, including age, histological type, histologic grade, tumour invasion, clinical stage, primary therapy outcome and lymphatic metastasis. Taken together, these data above clearly demonstrated that MCM10 changed in the genome, mRNA and protein levels of UCEC, but the effects of these changes on UCEC development still remains unclear.

In order to further explore the effects of MCM10 changes on UCEC, DEGs analysis based on TCGA database was used to enrich the underlying pathways. Subsequently, the results of gene function enrichment were accordant with the previous studies, which indicated that MCM10 positively regulated replication initiation,^26^ cell cycle^27^ and DNA repair.^28^ It is well known that the unlimited DNA replication is the basic characteristic of tumour cell proliferation, which closely depends on the cell cycle process.^29^ Therefore, high expression of MCM10 might contribute to the cell proliferation in UCEC, and this hypothesis was further verified by our results that the mRNA expression levels of most of MCMs, including MCM10, were positively associated with the expression levels of MKI67 mRNA in UCEC. MKI67 is a nuclear protein expressed in proliferating mammalian cell, which is one of the most widely used markers of proliferation in oncology and widely used as a proliferation indicator in the clinic.^30^, ^31^, ^32^, ^33^ In UCEC, MKI67 has been found to be related to the proliferation and invasion of UCEC cells. Thus, the regulation effect of MCM10 on UCEC could be revealed by its correlation with MKI67. Furthermore, our in vitro study indicated that blocking MCM10 was an effective strategy to inhibit UCEC cell proliferation and MKI67 expression, which implied that MCM10 targeting treatment might be beneficial for UCEC patients. However, it still needs more experiments to testify whether these effects were mediated by the inhibition of cell cycle or DNA replication. Moreover, DNA repair ensured the cells survival and led to chemotherapeutic resistance in tumour cells.^20^ In this regard, MCM10 blocking might induce cells into a state of DNA repair defect, which might be beneficial for chemotherapy‐resistant UCEC patients.

In this study, we found that the results obtained by using TCGA database to analyse the correlation between MCM10 mRNA and UCEC clinical characteristics were inconsistent with those obtained by using immunohistochemistry to analyse the correlation between MCM10 protein and UCEC clinical characteristics. The bioinformatic analysis showed that MCM10 mRNA expression was closely related to the age, histological type, histologic grade and clinical stage of UCEC patients. But the results form immunohistochemistry suggested that the expression of MCM10 protein was closely related to clinical stage and tumour invasion of UCEC patients. The inconsistency of the results might be related to the following reasons: mRNA expression abundance does not necessarily have a linear relationship with the protein expression level of its translation product, because protein content is regulated at many levels. Transcriptional regulation, post‐transcriptional regulation, translation and post‐translational regulation all play important roles in the final protein expression level. In addition, mRNA degradation, protein degradation and modification folding may lead to mRNA abundance and protein expression levels are inconsistent. All of these factors would cause the mRNA expression level to be inconsistent with the protein expression level, leading to the difference in the final analysis results.^34^

Our results indicated that both protein and mRNA levels of MCM0 increased in advanced UCEC, which suggested that MCM10 could be a treatment target for advanced UCEC. Recently, immune checkpoint inhibitors combined with small molecule inhibitors have achieved encouraging results in the treatment of advanced or relapsed UCEC. Pembrolizumab was originally used to treat UCEC patients with mismatch repair defects (MMRd). It was inspired that pembrolizumab in combination with lenvatinib, a small molecule multikinase inhibitor, was given approval by FDA for advanced or relapsed UCEC patients with MMR‐proficient due to well response rate of this combination therapy.^2^ Considering that MCM10 was closely related to DNA repair, we prudently suspected that MCM10 inhibitors could be used to broaden the indications for some medications in the treatment of advanced or relapsed UCEC.

Increasing studies revealed that some small molecule inhibitors could regulate the immune microenvironment of tumour tissue and promote immune‐mediated tumour cell death.^35^ Our results of gene function enrichment showed that MCM10 changes might influence the UCEC immune microenvironment, including humoral immune response, complement cascade and secreted factors. Furthermore, our results revealed that the expression of MCM10 mRNA was positively (negatively) correlated with the proportion of some immune cells. Infiltrating immune cells are an important part of the tumour immune microenvironment, and these cells might be conducive or block tumour development.^36^ The transition from Th1/Th2 balance to Th2 dominance was an obstacle of antitumor immunotherapy. Restoring the Th1/Th2 balance was of great significance in the treatment of tumour.^37^, ^38^ NK CD56 bright cell is an immature NK cell subset exerting anti‐tumour effect.^39^ Meanwhile, cancer patients showed a hypofunctional state of tumour‐infiltrating pDC cells.^40^ Taken together, using the property that MCM10 might increase the proportions of Th2 cells and decrease the proportions of NK CD56 bright and pDC cells, it could be attempted to combine MCM10 inhibitors with immune checkpoint inhibitors in the future to reprogram the tumour immune environment and further enhance the effects of immunotherapy for UCEC patients.

In recent years, based on tumour microenvironment‐ and immune‐related genes, several models predicting the prognosis of endometrial cancer have been constructed.^41^, ^42^ Considering that MCM10 was identified as a tumour immune microenvironment‐related gene and an independent risk factor for the OS of UCEC, TCGA data‐based prognosis prediction model for UCEC, incorporating MCM10 expression and some other independent risk factors, was firstly established in this study. Importantly, this model was then validated in our clinical data‐based prognosis prediction model. According to the expression of MCM10 and the independent risk factors from conventional clinical data, both models could be used to predict the individual 4‐, 6‐ and 8‐year OS probability. This novel strategy could be valuable in designing individualized long‐term follow‐up protocols and therapeutic regimes.

The shortcomings of our study are as follows: (i) Although we had validated the expression of MCM10 protein in our clinical samples, the sample size was small, and further validation with larger sample size are still required; (ii) the effects of MCM10 on the development of UCEC were just validated by the experiments in vitro, the experiments in vivo are needed for further validation in the future; (iii) the prediction model was only validated internally in this study and should be further validated externally using clinical specimens and data from other centres in the future.

### 4.1 Conclusions

In conclusion, based on public databases and our clinical data, we identified that MCM10 expression was significantly related to the development of UCEC. The function of MCM10 might be mainly involved in DNA replication, cell cycle, DNA repair and tumour immune microenvironment. By integrating TCGA and our clinical data, two novel prognostic models for UCEC were established, which could be used to effectively screen patients who need intensive follow‐up and intervention.

## AUTHOR CONTRIBUTIONS

**Junyu Chen:** Conceptualization (supporting); formal analysis (lead); methodology (lead); software (lead); visualization (lead); writing – original draft (lead). **Shan Wu:** Data curation (supporting); formal analysis (equal); methodology (equal); visualization (equal). **Junwei Wang:** Investigation (supporting); software (lead); supervision (lead); validation (lead); writing – review and editing (supporting). **Chunying Han:** Investigation (supporting); methodology (supporting); resources (equal); software (equal); validation (supporting); writing – review and editing (supporting). **Lijing Zhao:** Conceptualization (supporting); methodology (supporting); resources (equal); validation (supporting); writing – review and editing (equal). **Kang He:** Investigation (supporting); methodology (supporting); resources (supporting); visualization (supporting); writing – review and editing (supporting). **Yan Jia:** Conceptualization (equal); funding acquisition (equal); project administration (equal); supervision (equal); writing – review and editing (lead). **Manhua Cui:** Conceptualization (lead); funding acquisition (lead); project administration (lead).

## FUNDING INFORMATION

This research was funded by the National Natural Science Foundation of China, grant number: 81772772; Jilin Scientific and Technological Development Program, grant number: YDZJ202201ZYTS580, YDZJ202302CXJD062.

## CONFLICT OF INTEREST STATEMENT

The authors declare that they have no competing interests.

## PATIENT CONSENT STATEMENT

The patients were informed and agreed to participate in this study.

## Supporting information


Figure S1:

Figure S2:

Figure S3:

Figure S4:

Table S1:

Table S2:

Table S3:

Table S4:

Table S5:

Table S6:

Table S7:

Table S8:

Table S9:

Table S10:

Table S11:

## Data Availability

The data sets generated during and/or analysis during the current study are available from the corresponding author on reasonable request.
