# Supplementary figures and images for: MCM10: An effective treatment target and a prognostic biomarker in patients with uterine corpus endometrial carcinoma
Source: J Cell Mol Med. 2023 May 29;27(12):1708–24. doi: 10.1111/jcmm.17772 (PMC10273062; doi:10.1111/jcmm.17772)

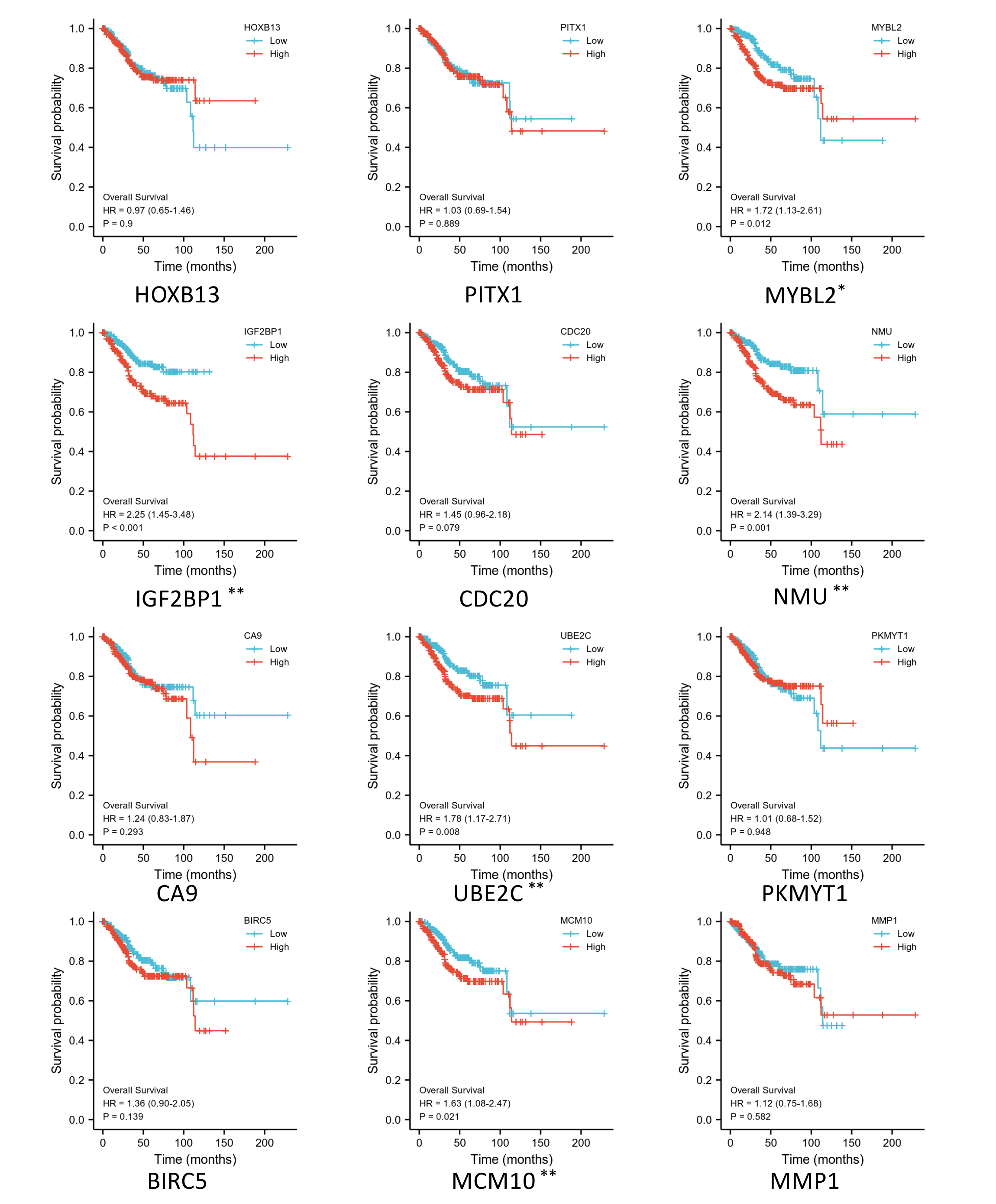

Supplement: Supplementary file 1 — Figure S1: [file JCMM-27-1708-s002.tif]

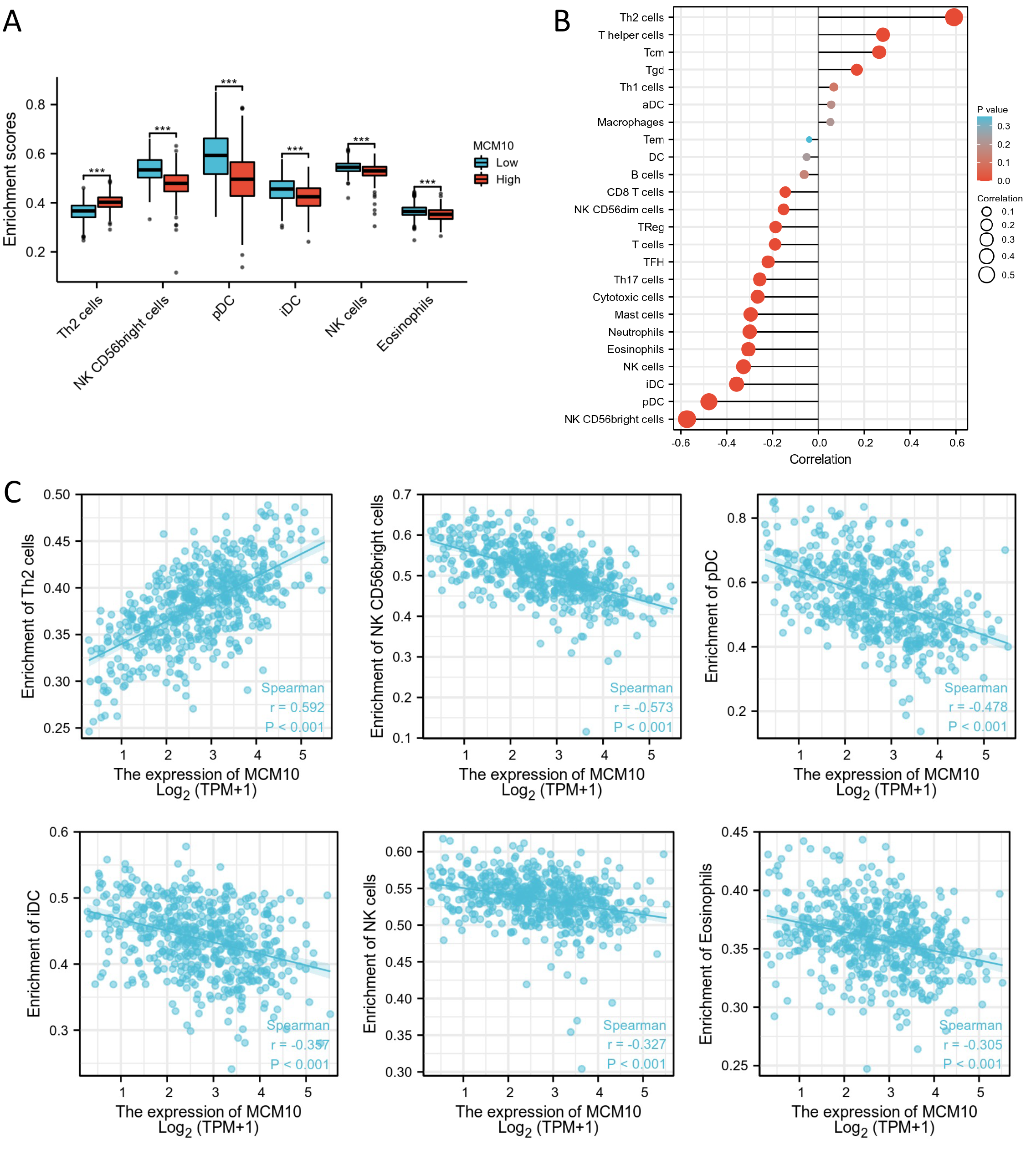

Supplement: Supplementary file 2 — Figure S2: [file JCMM-27-1708-s008.tif]

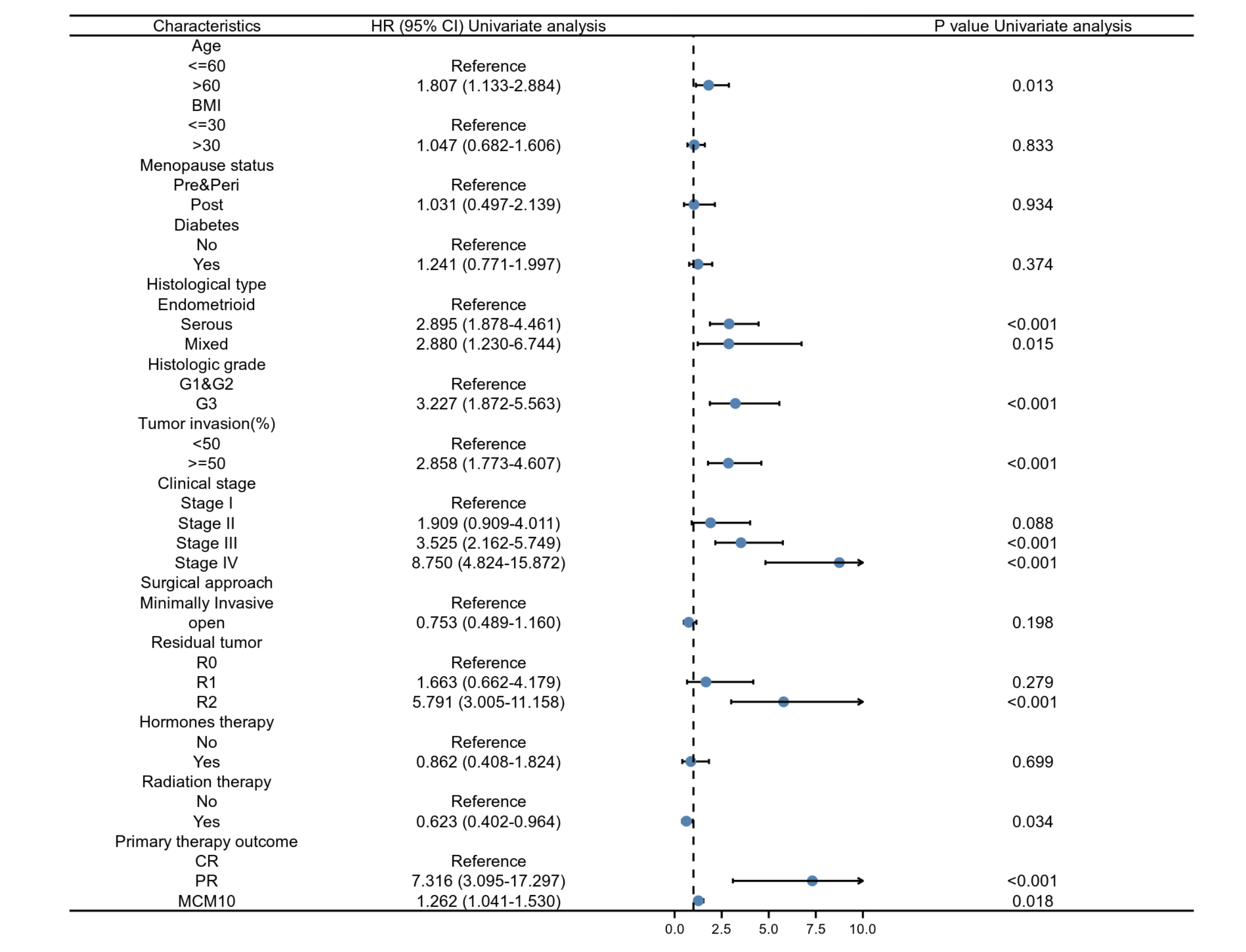

Supplement: Supplementary file 3 — Figure S3: [file JCMM-27-1708-s015.tif]

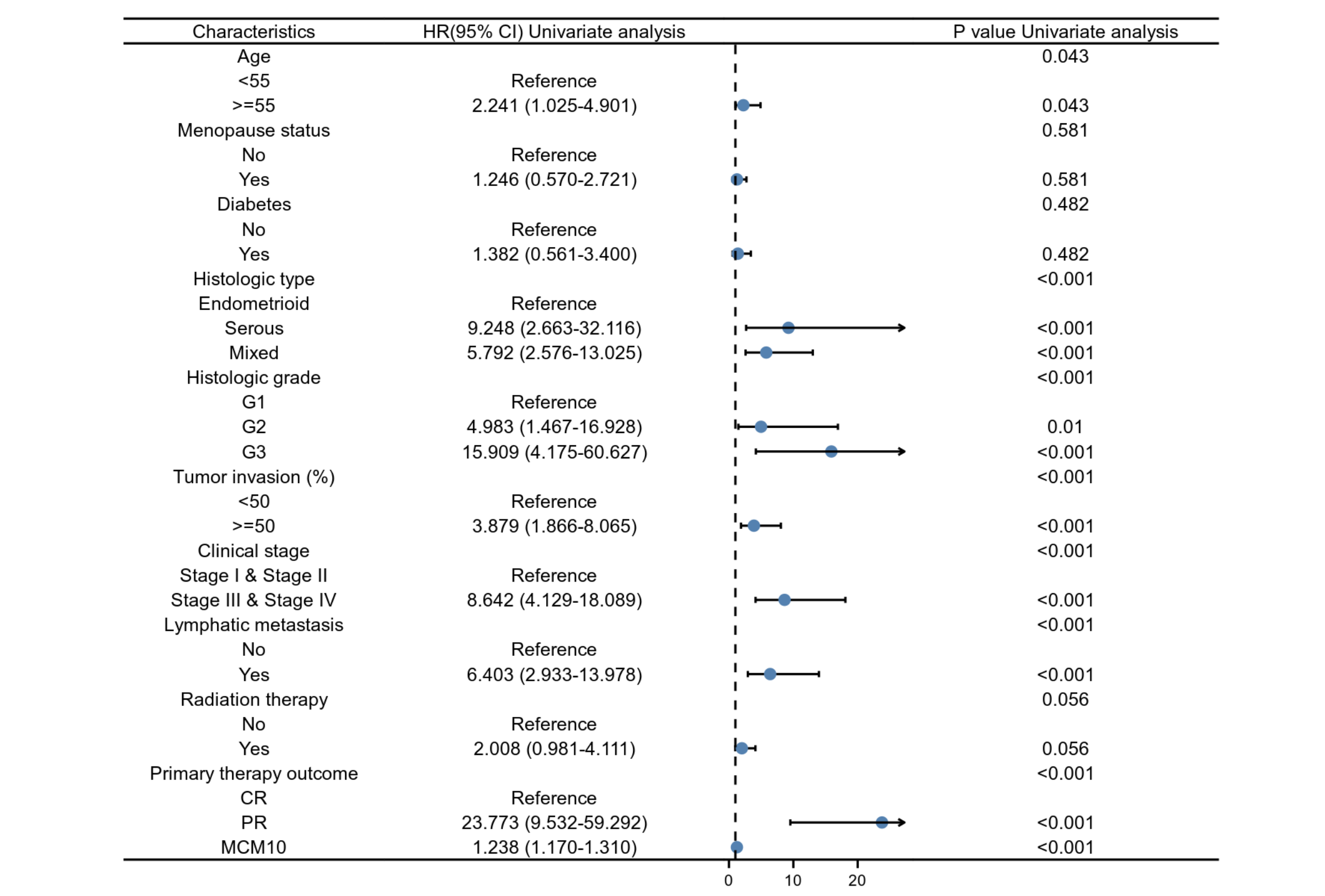

Supplement: Supplementary file 4 — Figure S4: [file JCMM-27-1708-s010.tif]
