# Supplementary material for: MCM10: An effective treatment target and a prognostic biomarker in patients with uterine corpus endometrial carcinoma
Source: J Cell Mol Med. 2023 May 29;27(12):1708–24. doi: 10.1111/jcmm.17772 (PMC10273062; doi:10.1111/jcmm.17772)
Supplement: Supplementary file 15 — Table S11: [file JCMM-27-1708-s014.docx]

| **Clinical features**  Association between MCM10 mRNA expression and clinical features of patients with UCEC | **Number of cases** | **Low expression of MCM10, number (%)** | **High expression of MCM10, number (%)** | **p** |
| --- | --- | --- | --- | --- |
| **Age, n (%)** **^a^** |  |  |  | 0.054 |
| <=60 | 206 | 115 (20.9%) | 91 (16.6%) |  |
| >60 | 343 | 161 (29.3%) | 182 (33.2%) |  |
| **Menopause status, n (%) ^a^** |  |  |  | 0.956 |
| Pre | 35 | 17 (3.4%) | 18 (3.6%) |  |
| Peri | 17 | 9 (1.8%) | 8 (1.6%) |  |
| Post | 454 | 225 (44.5%) | 229 (45.3%) |  |
| **Diabetes, n (%)^a^** |  |  |  | 0.425 |
| No | 328 | 163 (36.1%) | 165 (36.6%) |  |
| Yes | 123 | 67 (14.9%) | 56 (12.4%) |  |
| **Histological type, n (%)^a^** |  |  |  | **< 0.001^**^** |
| Endometrioid | 410 | 234 (42.4%) | 176 (31.9%) |  |
| Mixed | 24 | 9 (1.6%) | 15 (2.7%) |  |
| Serous | 118 | 33 (6%) | 85 (15.4%) |  |
| **Histologic grade, n (%)^a^** |  |  |  | **< 0.001^**^** |
| G1 | 98 | 83 (15.3%) | 15 (2.8%) |  |
| G2 | 120 | 80 (14.8%) | 40 (7.4%) |  |
| G3 | 323 | 111 (20.5%) | 212 (39.2%) |  |
| **Tumor invasion (%), n (%) ^a^** |  |  |  | 0.084 |
| <50 | 259 | 147 (31%) | 112 (23.6%) |  |
| >=50 | 215 | 104 (21.9%) | 111 (23.4%) |  |
| **Clinical stage, n (%)^a^** |  |  |  | **< 0.001^**^** |
| Stage I | 342 | 196 (35.5%) | 146 (26.4%) |  |
| Stage II | 51 | 24 (4.3%) | 27 (4.9%) |  |
| Stage III | 130 | 45 (8.2%) | 85 (15.4%) |  |
| Stage IV | 29 | 11 (2%) | 18 (3.3%) |  |
| **Primary therapy outcome, n (%) ^b^** |  |  |  | 0.170 |
| PD | 20 | 8 (1.7%) | 12 (2.5%) |  |
| SD | 6 | 3 (0.6%) | 3 (0.6%) |  |
| PR | 12 | 3 (0.6%) | 9 (1.9%) |  |
| CR | 442 | 236 (49.2%) | 206 (42.9%) |  |

1. the results were analyzed by Chisq. test.
2. the results were analyzed by Fisher. test.

_**_p < 0.01
